# A Large Gastrointestinal Tumor With Cystic Degeneration Initially Mistaken for Gastric Splenosis: A Case Report

**DOI:** 10.7759/cureus.94318

**Published:** 2025-10-10

**Authors:** Kelly Chan, Chi Yeung Lam, Lawrence Lai

**Affiliations:** 1 Gastroenterology and Hepatology, Pok Oi Hospital, Yuen Long, HKG

**Keywords:** abdominal splenosis, endoscopic ultrasound (eus), gastric submucosa, gastrointestinal stromal tumor (gist), submucosal tumor

## Abstract

Gastrointestinal stromal tumors (GISTs) are the most common mesenchymal neoplasms of the gastrointestinal tract and usually present as submucosal gastric lesions. Atypical morphologies, such as exophytic or cystic growth, are uncommon and can lead to lesions being mistaken for hepatic, pancreatic, or splenic origin. We report a case of a 63-year-old woman referred for evaluation of unexplained weight loss. Imaging revealed a 4 cm lobulated lesion adjacent to the spleen with peripheral cystic changes. The mass enhanced similarly to splenic tissue and was initially interpreted as splenosis. Final pathology revealed a GIST with cystic degeneration, negative margins, and no evidence of metastasis. This case highlights the diagnostic challenges posed by atypical GISTs and emphasizes the importance of endoscopic ultrasound (EUS) with tissue acquisition for accurate diagnosis when cross-sectional imaging is inconclusive.

## Introduction

Gastrointestinal stromal tumors (GISTs) are mesenchymal neoplasms of the gastrointestinal (GI) tract, representing approximately 80% of GI mesenchymal tumors [1]. They most commonly arise in the stomach, and less commonly from the small bowel, colon, rectum, and esophagus. GISTs originate from the interstitial cells of Cajal or their precursors and are typically characterized by mutations in the KIT or PDGFRA genes. Diagnosis is established through histopathological and immunohistochemical analysis, with CD117 (c-KIT) and DOG1 serving as key diagnostic markers. Management usually involves complete surgical resection for localized disease, with tyrosine kinase inhibitors, such as imatinib, for unresectable or metastatic tumors. While the majority of GISTs present as solid, well-defined, intramural masses, atypical presentations have been reported. Extraluminal or cystic variants of GIST are uncommon and may mimic a variety of benign and malignant pathologies, such as pancreatic cystic lesions, gastric duplication cysts, or gastric splenosis [2].

We present a case of a middle-aged woman with an atypical GIST that exhibited an exophytic, predominantly cystic morphology and was located adjacent to the spleen. Given its imaging features and anatomical position, the lesion was initially mistaken for gastric splenosis. Further evaluation with endoscopic ultrasound (EUS) revealed a mixed cystic-solid mass, and fine-needle biopsy (FNB) confirmed the diagnosis of GIST.

This case highlights the diagnostic challenges encountered in GISTs with atypical morphology or location, underscoring the importance of maintaining a broad differential diagnosis for cystic upper abdominal masses. It also emphasizes the value of early EUS assessment and tissue acquisition through FNB in achieving accurate diagnosis and guiding appropriate management.

## Case presentation

A 63-year-old East Asian woman with no significant past medical or surgical history was referred to our unit for evaluation of unintentional weight loss over several months. She denied abdominal pain, early satiety, gastrointestinal bleeding, or constitutional symptoms apart from mild fatigue. Physical examination was unremarkable, with no palpable abdominal mass, organomegaly, or lymphadenopathy.

Initial chest imaging performed during her evaluation revealed a 1.5 cm left apical lung nodule. To further characterize this finding, a whole-body positron emission tomography-computed tomography (PET-CT) scan was undertaken. Incidentally, the scan demonstrated a 4 cm lobulated soft-tissue lesion situated anterior-inferior to the spleen, exhibiting peripheral cystic changes and enhancement patterns similar to splenic tissue. The lesion showed no significant fluorodeoxyglucose (FDG) uptake, leading to a radiological impression of splenosis.

**Figure 1:**
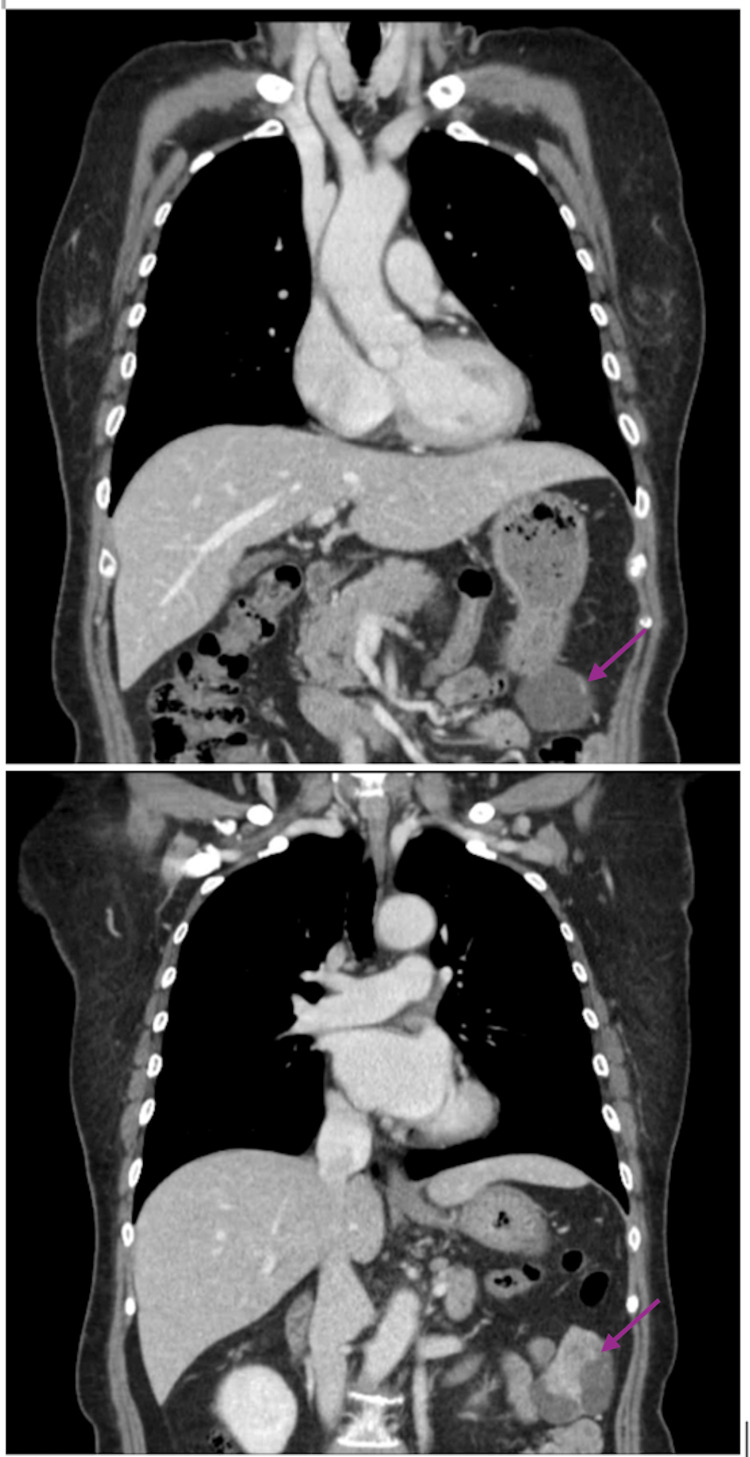
Coronal section of CT showing a mixed cystic-solid lesion (arrows) which appears to arise from the greater curve of the stomach.

On further review, the patient denied any history of abdominal trauma, surgery, or splenic disease. Laboratory investigations were within normal limits. Serial thoraco-abdominal CT imaging over the following six months demonstrated no interval change in the lesion. Upper endoscopy performed for malignancy screening failed to identify a corresponding intraluminal lesion, and the working diagnosis of splenosis was maintained. Subsequent CT half a year later revealed persistence of the lesion, which appeared to arise from the gastric greater curvature (Figure 1).

**Figure 2:**
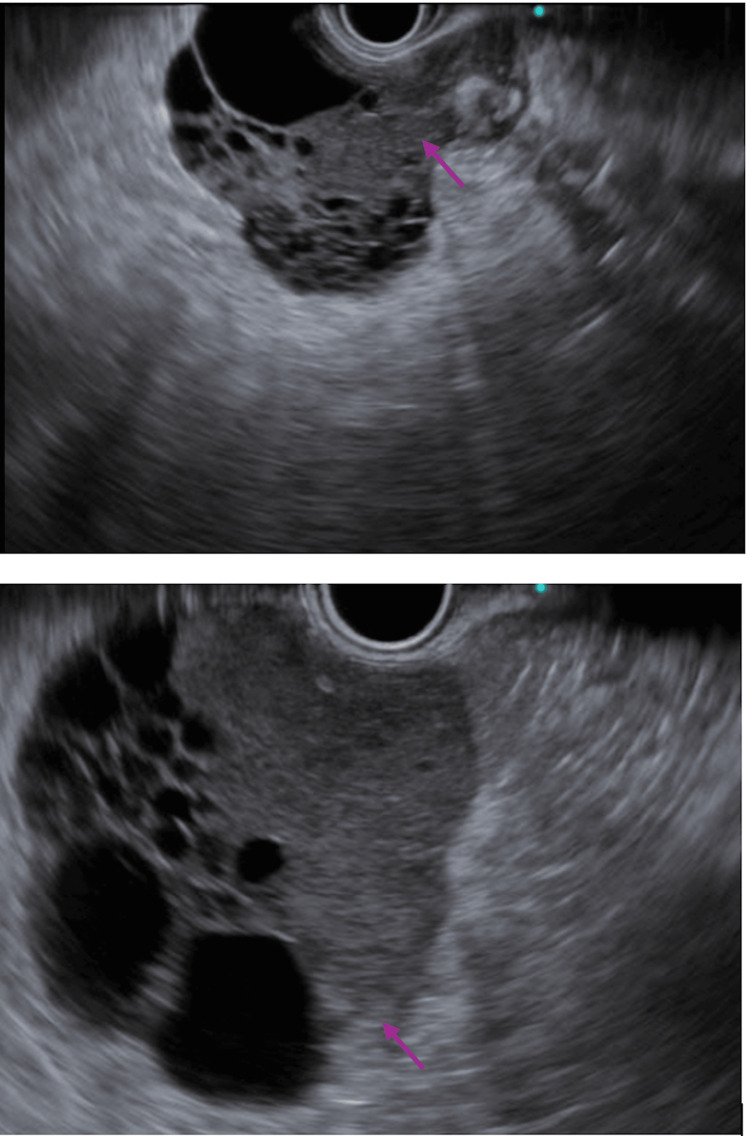
Endoscopic ultrasound demonstrated a 4×3 cm heterogeneous hypoechoic multilobulated lesion (arrows).

**Figure 3:**
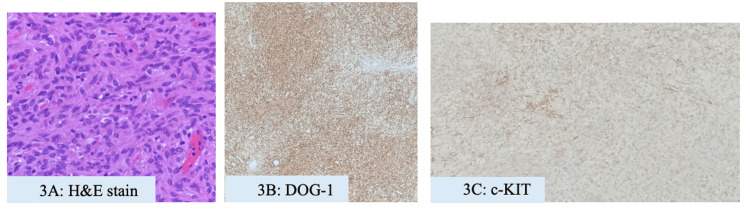
Histology immunohistochemistry findings of the patient. Histological findings of GIST showed spindle cells under H&E staining (A). Immunohistochemistry showed positivity for DOG1 (B) and c-KIT (C). GIST: gastrointestinal stromal tumor

Endoscopic ultrasound (EUS) was arranged and demonstrated a 4×3 cm heterogeneous hypoechoic multilobulated lesion, 50 cm from the incisors, with hyperechoic foci, acoustic shadowing, and internal calcification. The mass appeared to arise from the muscularis propria (Figure 2). Fine-needle biopsy (FNB) yielded spindle cell tissue arranged in fascicles, with uniform spindle-to-round nuclei, fine chromatin, and indistinct nucleoli. No tumor necrosis was observed. Immunohistochemistry showed positivity for DOG1 and c-KIT, consistent with GIST (Figures 3A-3C).

**Figure 4:**
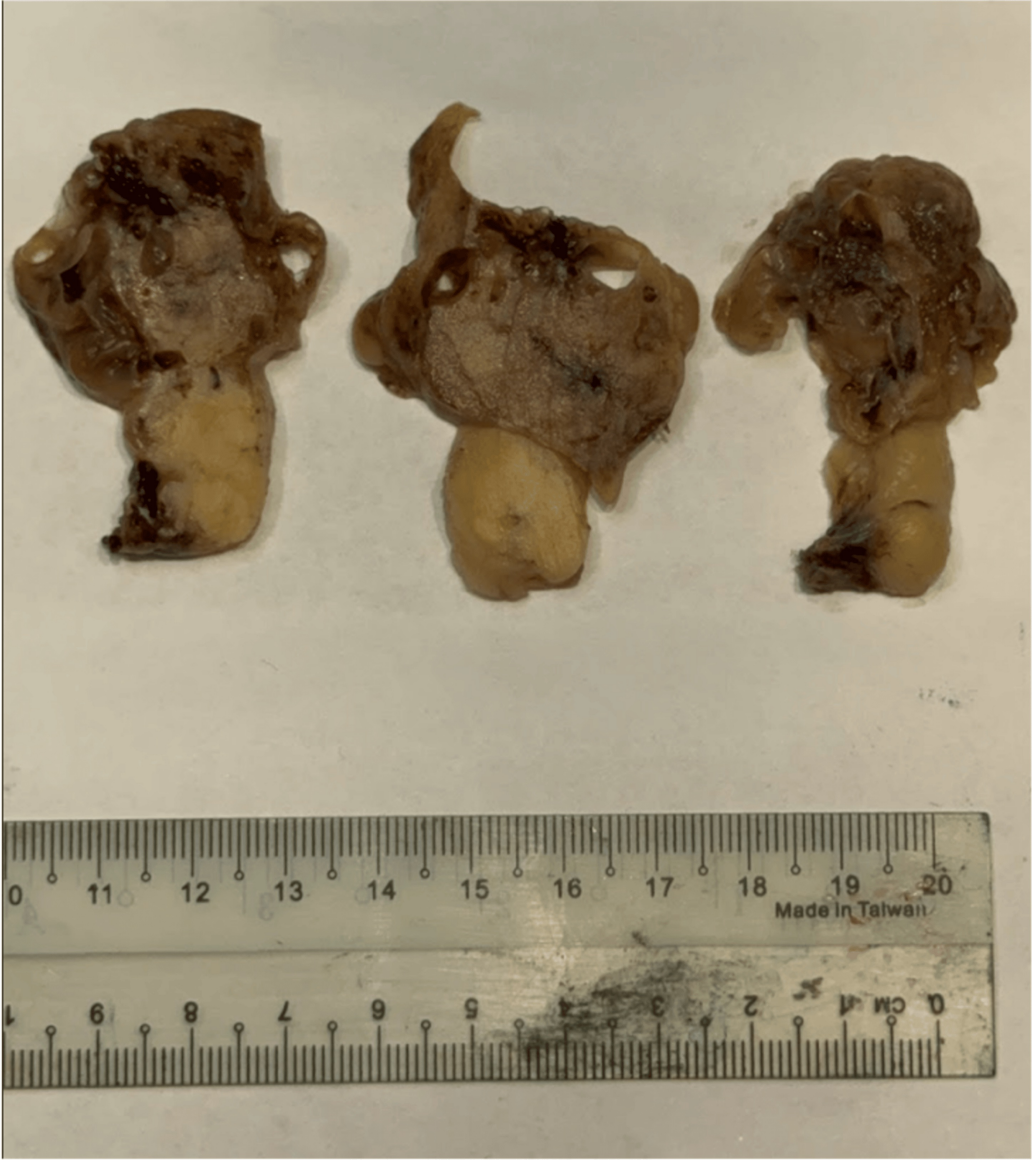
Gross pathology revealed a 6.5×5.5×4 cm multiloculated cystic mass originating from the gastric muscularis propria.

The patient underwent laparoscopic wedge resection. Intraoperatively, a 3 cm solid mass with a 5 cm cystic component on a stalk arising from the posterior wall of the lesser curvature was identified and resected en bloc. Gross pathology revealed a 6.5×5.5×4 cm multiloculated cystic mass originating from the gastric muscularis propria, with smooth serosal covering (Figure 4).

Gross pathology demonstrated cystic degeneration with focal hemorrhage. Histologically, the tumor had a mitotic index of one per 10 high-power fields and a Ki-67 proliferative index of 1%. Resection margins were clear. Cystic degeneration, hemorrhage with fibrin deposition, and brownish refractile pigment were noted. The risk of progressive disease was classified as low (3.6%) according to the College of American Pathologists (CAP) protocol.

The patient underwent an uneventful recovery and was discharged. Given the complete resection of the lesion and its low-risk pathological features, adjuvant therapy was not indicated. She was subsequently enrolled in a structured program of regular clinical and radiological surveillance. At her most recent follow-up four months post-operation, she remained asymptomatic. This case highlights the excellent prognosis of low-risk GISTs following complete resection and the importance of continued surveillance to detect potential late recurrence.

## Discussion

GISTs are mesenchymal neoplasms of the gastrointestinal tract that arise from the pacemaker cells of the muscularis propria, the interstitial cells of Cajal [3]. They most commonly present as solid masses. Diagnosis is typically established through a multimodal approach, incorporating endoscopy and histopathological evaluation, including immunohistochemistry (CD117 antigen expression) and molecular testing for activating mutations in KIT and PDGFRA [4].

Risk stratification is based on tumor size, anatomical location, and mitotic index, which allows classification into low- or high-grade lesions. Rare morphologic variants, such as pedunculated or cystic GISTs, may mimic lesions of hepatic, pancreatic, or splenic origin and are at risk of misdiagnosis, such as splenosis [5].

While GISTs are usually solid, well-defined, intramural masses in the stomach, atypical presentations of GIST (such as exophytic or cystic lesions in various case reports) can complicate diagnosis and mimic other abdominal masses. Zhu et al. noted a large cystic-solid mass arising from the pancreatic body in close proximity to the stomach. It was initially mistaken to be a pancreatic cystadenocarcinoma or canceration of cystadenoma. Surgical resection was arranged and histological results later showed a malignant GIST [6]. In another case, Naitoh et al. reported a multicystic tumor with extramural compression of the greater curvature of the antral stomach by the tumor, but EUS failed to demonstrate continuity to the stomach. Histological results also showed a low-grade GIST [7].

These cases, along with our own experience, underscore the diagnostic challenges posed by GISTs with unusual morphology and highlight the importance of early tissue acquisition via EUS-FNB for definitive diagnosis. Importantly, pedunculated morphology and cystic degeneration are often associated with aggressive disease [8], reflecting underlying processes, such as necrosis, intratumoral hemorrhage, and inadequate vascularization; hence, prompt diagnosis is necessary [9].

This case highlights the diagnostic importance of early evaluation with EUS tissue acquisition via fine-needle biopsy when suspicious lesions are detected on cross-sectional imaging [10]. It further illustrates the value of FNB in characterizing GISTs with high-risk sonographic features. Notably, certain GISTs may lack intraluminal growth and instead present in close proximity to adjacent organs, such as the spleen. In such scenarios, a presumptive diagnosis should not be made solely on anatomical location or imaging enhancement characteristics, but rather requires histopathological confirmation.

## Conclusions

This case demonstrates the diagnostic complexity of GISTs with atypical morphology, particularly when they present as exophytic or cystic masses in proximity to other abdominal organs. In this patient, the lesion was initially misinterpreted as splenosis due to its enhancement pattern and location near the spleen, highlighting the limitations of relying solely on cross-sectional imaging for diagnosis.

Our experience reinforces the value of early EUS with FNB for tissue acquisition, which enables definitive diagnosis and accurate risk stratification. While cystic degeneration in GISTs is often associated with aggressive biological behavior, this case underscores that low-risk variants can also display atypical features. Ultimately, histopathological confirmation remains the gold standard for diagnosis, ensuring patients receive appropriate treatment and follow-up surveillance. Clinicians should maintain a high index of suspicion for GIST and obtain early histological evaluation when assessing sub-epithelial gastric or perisplenic lesions with atypical morphology, as delayed diagnosis may result in disease progression and an increased risk of metastasis, potentially compromising clinical outcomes.
